# Physiology of microalgal biofilm: a review on prediction of adhesion on substrates

**DOI:** 10.1080/21655979.2021.1980671

**Published:** 2021-10-04

**Authors:** Yi Tong Cheah, Derek Juinn Chieh Chan

**Affiliations:** School of Chemical Engineering, Engineering Campus, Universiti Sains Malaysia, Nibong Tebal, Penang, Malaysia

**Keywords:** Biofilm, adhesion, microalgae, extracellular polymeric substances (EPS), cell-substratum interaction

## Abstract

In view of high energy cost and water consumption in microalgae cultivation, microalgal-biofilm-based cultivation system has been advocated as a solution toward a more sustainable and resource friendlier system for microalgal biomass production. Algal-derived extracellular polymeric substances (EPS) form cohesive network to interconnect the cells and substrates; however, their interactions within the biofilm are poorly understood. This scenario impedes the biofilm process development toward resource recovery. Herein, this review elucidates on various biofilm cultivation modes and contribution of EPS toward biofilm adhesion. Immobilized microalgae can be envisioned by the colloid interactions in terms of a balance of both dispersive and polar interactions among three interfaces (cells, mediums and substrates). Last portion of this review is dedicated to the future perspectives and challenges on the EPS; with regard to the biopolymers extraction, biopolymers’ functional description and cross-referencing between model biofilms and full-scale biofilm systems are evaluated. This review will serve as an informative reference for readers having interest in microalgal biofilm phenomenon by incorporating the three main players in attached cultivation systems: microalgae, EPS and supporting materials. The ability to mass produce these miniature cellular biochemical factories via immobilized biofilm technology will lay the groundwork for a more sustainable and feasible production.

## 1. Introduction

In many developing countries, intensive agricultural activities and rapid industrialization have led to the rise in wastewater production and the scarcity of clean water supply. In view of regulating the concentration of pollutants in the discharging effluent, algae-based bioremediation technologies provide a compelling solution due to their great adsorption capacity of inorganic nutrient and effective fixation of inorganic compounds, including carbon dioxide and heavy metals [1]. The appeal of microalgal culture utilization stems from the fact that conventional treatment systems by activated sludge mainly prioritize the eradication of solid suspension and the reduction in biochemical oxygen demand [2]. In addition, such systems have drawbacks, including high operational costs, large operating foot print, underutilized natural resources, secondary pollution from chemical processes, and immense potential for carbon dioxide emission over time during storage [1]. Meanwhile, realizing the rising dependence of sustainable alternative energy sources to fulfill the needs of growing population, microalgae are also recognized as one of the most promising feedstocks of biofuels and bio-functional chemicals. Success stories of bio-ethanol, bio-methane and bio-hydrogen production via palm oil mill effluent treatment using microalgae to address the global energy security concern have been reported [3].

Microalgae are typically grown in open systems such as open raceway ponds or closed systems like photo-bioreactors (PBR), where the cells are suspended in liquid medium, but in a more well-controlled environment. Nevertheless, such microalgae cultivation system designs are facing the same bottleneck, whereby the biomass productivities are relatively low. The cell concentrations in open ponds could be as low as 0.5 g/L (0.05% dry basis) and 2–6 g/L (0.2–0.6% dry basis) in photobioreactors [4]. Moreover, the harvesting of microalgal biomass from the diluted broth is normally performed through sedimentation, flocculation, flotation or centrifugation. Multiple operations involved to harvest the biomass are time-consuming and cost-ineffective. Surging demands of microalgae biomass have motivated a large number of researches aiming to improve the traditional culture systems, and this eventually leads to a state-of-the-art cultivation technology of immobilized biofilm cultivation [5]. This type of culture system has jotted down numerous advantages over the conventional systems, such as less water and energy requirement, high process controllability and high biomass productivity. By growing algae as biofilm attached firmly to solid substrates, there is huge potential to concentrate the harvested biomass from 0.4% in planktonic systems to 8–16% in biofilms [6,7]. Furthermore, a thin water film serving as a boundary layer between the biofilm and ambient gas phase allows for short gas diffusion pathways and minimizes hydrodynamic stress on the attached cells. The periodic liquid flow can constantly distribute both nutrients and gases across the entire biofilm surfaces [5].

However, as compared to suspended culture systems, there is still lack of industrial exposure for biofilm immobilized cultivation. Amidst the previous studies on microalgal biofilm, majority of the works revolved around the studies of cell attachment to various materials in lab scales [8,9]. It was a popular trend for researchers to investigate the influence of different environmental conditions, such as pH, temperature, light intensity and medium flow rate toward the biofilm development onto different substrates [8]. In addition, some remarkable studies have been conducted to understand the impact of substrate surface properties, such as roughness and hydrophobicity onto microalgal biofilm formation to verify the theoretical hypothesis [10,11]. By understanding the interaction between the microalgal biofilm and the substrates, these fundamental studies were able to provide insight into the ways of maximizing biofilm adhesion and biomass yield. There were some bench and pilot-scale studies on the construction of novel bioreactor systems with algal biofilms as the principal figure too to determine the lipid accumulation rates and biomass production rates [12,13]. Treating wastewater using microalgal biofilm has gained the most scientific interest, especially using algal turf scrubbers and rotating algal biofilm reactors [7,14].

Hence, the main purpose of this review attempts to summarize the current knowledge of microalgal biofilm adhesion and the biofilm composition changes when attached to different surfaces in marine or freshwater systems. In **Section 2**, different microalgal biofilm cultivation systems and biofilm adhesion strength with respect to substrate are discussed with the aid of illustration and supporting research data. **Section 3** elucidates the abiotic conditions that contributed to the changes in the biofilm composition. To predict the microalgal adhesion onto substrates with varying surface tensions, **Section 4** covers fundamental mathematical models that reveal the specific nature of cell adhesion mechanisms. This current work will foster the understanding of how the polymeric substances in biofilm affect the biofilm attachment and how they are supported by the mathematical inferences. Lastly, present challenges and future outlook are discussed in **Section 5** to highlight the potential of microalgal biofilm applications. Considering the emergence of this contemporary biotechnology, it will be beneficial to the research groups working on biofilm to work toward routes that not only promote sustainability of the designed system but also the valorization of microalgal metabolite production.

## 2. Biofilm adhesion onto different substrates

### 2.1. Biofilm matrix and extracellular polymeric substances (EPS)

Microalgal biofilm develops on any illuminated surfaces surrounding with available nutrients and it composes of entrapped unicells ranging in the size from a few to several hundred micrometers [15]. Biofilm is a highly structured and dynamic microalgal community in which the cells encase themselves in a matrix of self-produced EPS [16]. Typically, biofilm is always described as a tiny city for microalgal cells because the cells sustain themselves by uptaking the nutrient that are continuously diffuse in from the surrounding. Besides its protective function, the biofilm matrix keeps the extracellular enzymes close to the cells, allowing the metabolism to occur [17]. Biofilm formation process is species-dependent and complicated but can be basically divided into five main stages: (1) the floating cells attach to the substrates through adsorption to form a reversible conditioning film; (2) irreversible adhesion begins due to the secretion of EPS, which colonize the cells to the surface via hydrogen bonding; (3) organic molecules concentrates on the surfaces favor the replication of early colonizers; (4) biofilms gradually mature, raising its height into a three-dimensional (3D) structure with multiple layers; and (5) biofilms loss its integrity at a given thickness,especially during cell lysis [18].

Biofilm is made up of 90% of EPS and 10% algal cells. EPS are biomolecules and inert solids that group the cells and bind them to substrates [19]. They are actively produced by the trapped cells in the biofilm to the surrounding and commonly comprise proteins, phospholipids, polysaccharides, nucleic acids, humic substances, uronic acids and some functional groups, such as phosphoric, carboxylic, hydroxyl and amino groups [20]. EPS majorly consist of water and collapse when dehydrated, but it still constitute up to 90% of organic matter in the biofilms [21]. All classes of the macromolecules in EPS interact with each other and have their respective functions to maintain the stable 3D matrix. EPS not only serve as energy and carbon sinks to the cells but also safeguard the cells from dehydration [22]. Most importantly, these biomolecules reinforce the cell adhesion to substrates by interconnecting the cells and promoting ensheathment in the biofilm matrix. The microalgal cells living in EPS matrix creates a completely different lifestyle from the suspension state [23]. Since the distribution of these biomolecules greatly affected the biofilm structure; hence, it is crucial to understand the respective functions of the EPS components as presented in Table 1.

**Table 1.** Functions of related EPS components. (Adapted from [17,61])

EPS components	Functions
Polysaccharides	Enhances biofilm-substrate adhesion
	Aggregates microalgal cells
	Supplies nutrient for biofilm utilization
	Exhibits flow and elastic recovery to the matrix shape
	Forms a hydrated polymer network
	Enhances the tolerance of microenvironment in water-deficient environments
	Offers host defenses toward infection
	Stores excess carbon under unbalanced carbon to nitrogen ratios
	Accumulates enzymes
Proteins	Enhances biofilm-substrate adhesion
	Forms a hydrated polymer network
	Allows cell-cell communication
	Enhances the tolerance of microenvironment in water-deficient environments
	Offers host defenses toward infection
	Digests exogenous macromolecules for nutrient acquisition
	Degrades structural EPS, releasing cells from biofilms
Lipids	Bio-surfactant and bio-emulsifier
Nucleic acids (DNA)	Regulates the biofilm formation and structure
	Enhances biofilm-substrate adhesion
	Facilitates horizontal gene transfer between biofilm cells
	Aids metabolic turnover by exporting cell components
Uronic acids	Interact with cations to increase metal ions concentration for cells in oligotrophic condition
Humic substances	Permits redox activities in the biofilm
Sulfates	Impart EPS hydrophilicity to have gel-like consistency
Cations (Ca^2+^ and Mg^2+^)	Cross-links different polysaccharide chains

**Figure 1.**
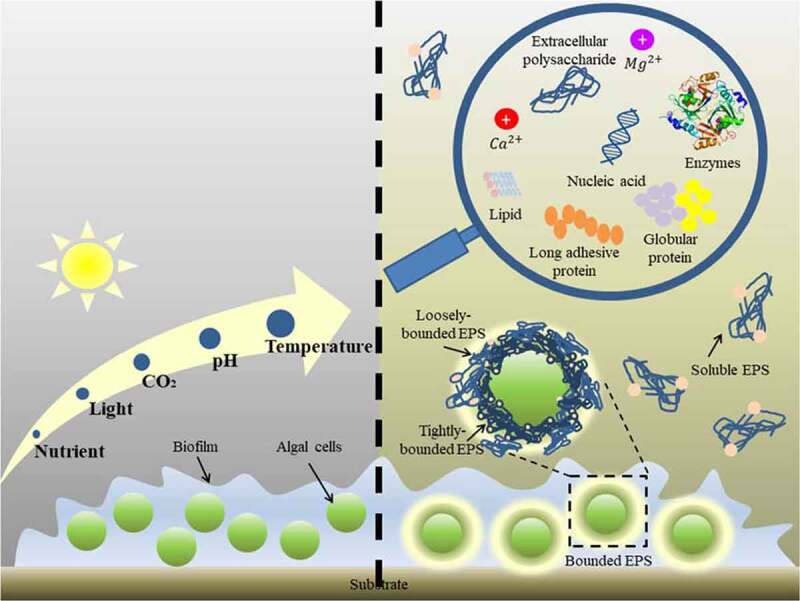
Schematic diagram of the microalgal biofilm and extracellular polymeric substance (EPS) structure. (Modified from [117])

As illustrated in Figure 1, EPS can be divided into two categories, soluble EPS (sEPS) and bounded EPS (bEPS). sEPS are distributed in the medium and bEPS are attached on the cells surfaces. sEPS can be sometimes resulted from the hydrolysis of bEPS. bEPS can be further subdivided into tightly bounded EPS (TB-EPS) and loosely bounded EPS (LB-EPS) [24]. Each type of EPS depicts unique composition and set of functionalities as LB-EPS showed the highest Pb(II) adsorption abilities as compared to TB-EPS and sEPS [25]. EPS composition varies with species, substrate type, nutrient availability, temperature, pH and light intensity. As summarized by Babiak et al. (2021), polysaccharides account for 45–95% of total EPS, while total extracellular protein amount varies in a range from 0.5% to 42.1% in green microalgae, diatoms and red algae [26]. Indeed, the protein content from microalgae cultivated in manure wastewater was 51.6%, which was 7.5% greater than that of microalgae cultivated in culture medium. Microalgae grown in wastewater, on the other hand, had lipid content that was 5–15% lower than that of culture medium [13]. In contrast, total polysaccharide, protein and lipid content extracted from benthic diatom, *Nitzschia* sp. cultured in 100% sea cucumber aquaculture wastewater were significantly higher than that of growth medium due to the accumulation of nitrogen and phosphorus from wastewater [27]. These varying EPS compositions aid in defining the characteristics of each EPS substituents as adhesiveness substantially depends on the chain conformation and internal/external substituents interactions. Therefore, the extent to which type of biomolecules contributes the most to cell adhesion remains to be investigated.

From the nature of individual components of EPS, the formation and maintenance of a biofilm matrix totally depends on the EPS production. A number of extensive researches were done on quantification of microalgal EPS production, but the physic-chemical property between the EPS and cells is yet to be explored. To date, many researchers have been focusing their efforts on characterization and performance of algal biofilm for industrial applications. The contribution of those polymers to the biofilm bonding strength is poorly understood. Indeed, EPS production could be stimulated by different substrates used; therefore, more future researches are required to boost the understanding of the EPS toward the biofilm adhesion. New knowledge of biofilm adhesion will be able to predict the algal adhesion speed onto different substrates and to develop control strategies for microalgal attachment, for example, customizing a novel antifouling coating to prevent the biofouling that usually takes part in their natural environment.

### 2.2. Microalgal biofilm cultivation

Biofilm is always an indispensable characteristic in attached cultivation methods. Generally, microalgal biofilm can be categorized into two main types: single species biofilm for manufacturing purposes such as bio-transformations and multiple species that often found in waste removal areas such as wastewater treatments [28]. First type of biofilm is rare in nature but easier to be controlled for product optimization. In this context, less contamination was spotted because the whole cultivation process is mostly stable [29]. Hence, single-species biofilms are of great interest in biotechnology, although they rarely exist in nature. For instance, Shen et al. (2014) successfully investigated how culture period, culture volume, pH and initial total nitrogen concentration influenced the biomass productivity of single species, *Chlorococcum* sp adhered onto the glass fiber-reinforced plastic utilizing central composite design from response surface methodology toolkit [30]. When culturing mono-species biofilm, process parameters were easily regulated to allow a time-saving and cost-effective optimization by bringing down the chances of trial and error.

In the second-type multiple-species biofilm, there are interactions between the microalgae and bacteria ranging from mutualism to parasitism, which is governed by the secretion of organic matter released [31]. Transparent exopolymer particles (TEP) that are made up of the acidic sugar monomers secreted by microalgae will attract bacteria that provide important nutrients, such as iron, ammonium and vitamins into the biofilm [32]. Conversely, bacteria stimulate the TEP production from diatoms to serve as a new carbon source [33]. Some bacteria can even bring along hydrolytic exo-enzymes to break down large polysaccharides molecules released by microalgae [34]. Nevertheless, the interaction between them is still species-specific, thereby giving some compositional shifts when different diatom species are grown together. Koedooder et al. (2019) pointed out that the presence of bacteria decreased the productivity of diatom monocultures but did not seem to affect the diatoms growth in co-cultures. Instead, they have created a balanced micro-ecosystem by EPS combination [35]. This kind of biofilm that involves both autotrophic and heterotrophic components is suitable for wastewater treatment because it effortlessly uptakes the nutrients from polluted waters [29]. Moreover, multiple-species biofilm can easily regrow following a routine harvesting cycle, resulting in higher biomass productivity as the biofilm matures [36].

**Figure 2.**
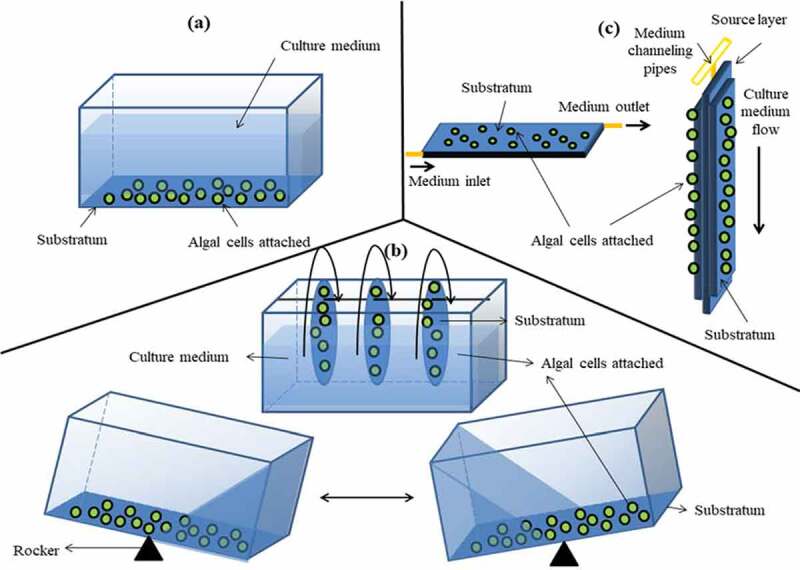
Schematic designs of the microalgal biofilm systems: (a) constantly submerged biofilms, (b) partially submerged biofilms and (c) permeated biofilms (adapted and modified from [83])

Microalgal biofilm is usually cultivated according to the application needs. As presented in Table 2 and Figure 2, biofilm cultivation system can be classified into three broad categories, which are constantly submerged biofilms, partially submerged biofilms and permeated biofilms. Various carrier materials have been studied for biofilm growth in recent years because each microalgae strain has its own adhesion affinity toward various substrates and behaves differently in their own EPS production. Substrate is normally chosen based on their availability, durability, reusability and pricing. Microalgal biofilm cultivation requires high integrity substrates such as polycarbonate and cellulose nitrate membranes as re-harvesting was performed frequently by scarping off the biomass from the surfaces. Nevertheless, the main drawbacks of submerged biofilm systems are high dissolved carbon dioxide requirement and escaping carriers due to wash out over time, while partially submerged biofilm systems are prone to mechanical failure due to constant mechanical movement over long duration, indirectly resulting in lower process flexibility. Though permeated biofilm systems offer extremely high oxygen transfer efficiency to the biofilm, but thicker biofilm impedes nutrient mass transfer, significantly upsetting the process especially when there is substrates defect. At present, huge-scale practical applications pertaining to microalgal biofilm systems are still rare, especially with the utilization of robust microporous substrates, which offer greater flexibility in biomass harvesting process.

**Table 2:** Comparison of microalgal biofilm cultivation on different substrates

Culture system	Cultivation area (m^2^)	Substrate	Temp (°C)	pH	Light attenuation (*µ* mol photons /m^2^/s) [light/dark (hr)]	Time (days)	Flow rate (L/min)	Rotation speed (rpm)	Culture medium	Species	Biomass productivity (g /m^2^/day)	Ref.
**Constantly submerged biofilms**												
Petri-dish biofilm	0.0004	Cotton	24 ± 2	6.5	90 ± 10 [24:0]	16	N/A	70	Bold’s basal	*S. vacuolatus*	[^b^ >60% at 2^nd^ hours]	[37]
		Jute fabrics									[^b^ >80% at 2^nd^ hours]	
Petri-dish biofilm	0.002	Polyvinylidene fluoride commercial membrane	25 ± 2	N/A	20.25 [12:12]	4	N/A	40	F/2	*Amphora coffeaeformis*	(^a^ ~2.84 x 10^8^ cells /m^2^ at 96^th^ hours)	[38]
										*Cylindrotheca fusiformis*	(^a^ ~1.33 x 10^8^ cells /m^2^ at 96^th^ hours)	
										*Navicula incerta*	(^a^ ~3.11 x 10^8^ cells /m^2^ at 96^th^ hours)	
Petri-dish biofilm	0.0004	Titanium	24 ± 1	6	60 [12:12]	2	N/A	40	Modified Chu 10	*Chlorella vulgaris*	(^a^ ~7.8 x 10^3^ cells /cm^2^ at 48^th^ hours)	[8]
										*Nitzschia amphibia*	(^a^ ~20 x 10^3^ cells /cm^2^ at 24^th^ hours)	
										*Chroococcus minutus*	(^a^ ~8 x 10^3^ cells /cm^2^ at 24^th^ hours)	
Curtain-biofilm in aquarium	N/A	Plastic net	20-22	N/A	260	5	N/A	N/A	F/2	*Navicula* sp.	(^a^ 1.24 x 10^9^ cells /m^2^ at 5^th^ days)	[9]
Multi-layers photobioreactor	0.007	Polyethylene foam	26 ± 2	N/A	100 ± 10 [12:12]	20	0.003	N/A	Modified basal	*Botryococcus braunii*	1.50	[13]
		Glass fiber reinforced plastic									3.20	
		Polyethylene foam							Piggery farm wastewater		3.71	
		Glass fiber reinforced plastic									2.91	
Flat plate photobioreactor (PBR)	0.004	Glass	26 ± 2	7	160 [16:8]	20	0.013-0.023	N/A	Modified Chu 10	*Nitzchia palea*	2.8	[74]
										*Scenedesmus obliquus*	2.1	
Membrane biofilm reactor	0.0017	Mixed cellulose esters membrane	26 ± 2	6.8-7.5	50 [14:10]	8	0.001	N/A	Culture medium (2 g/L glycerol)	*Chlorella vulgaris*	9.27	[39]
Attached cultivation system	0.005	Glass fibre-reinforced plastic	26 ± 2	7	150 [14:10]	14	N/A	N/A	Artificial seawater (18 mM glycine)	*Nannochloropsis oculata*	15.76	[40]
Attach cultivation system	N/A	Filter paper	20 ± 2	N/A	100	8	0.01	N/A	Swine wastewater	*Chlorella pyrenoidosa*	5.03	[41]
Algal biofilm photobioreactor	0.28	Flexible fiber bundles	25-28	6.8-7.5	8000 lux	20	0.07	N/A	Simulated secondary effluent	*Chlorella vulgaris*	0.05	[42]
**Partially submerged biofilms**												
Flat plate algal biofilm photobioreactor (FPBR)	0.015	Rice husk	25 ± 2	6.8	300	20	0.003	N/A	BG 11	*Scenedesmus obliquus, Chlorella vulgaris, Oscillatoria tenuis mixed culture*	7.32	[114]
		Pine sawdust									10.92	
		Oak sawdust									8.67	
		Sugarcane bagasse									9.54	
Rotating algal biofilm (RAB)	0.045	Cotton duct	25	N/A	110-120 [24:0]	7	N/A	4	Bold’s basal	*Chlorella vulgaris*	3.51	[43]
RAB	0.186	Cotton rope	19	N/A	170 [14:10]	12	N/A	4.8	Logan WWTP wastewater	Mixed culture of algal-bacterial	5.5	[7]
Rotating flat plate photobioreactor	0.036	PVC	22 ± 2	N/A	139 [12:12]	24		2.8	Bold’s basal	*Chlorella vulgaris SAG 211-12*	0.42	[12]
Algadisk rotating biological contactor	0.09	Rough stainless steel mesh	38 ± 1	6.75	422	7	N/A	11	M8-a	*Chlorella sorokiniana*	20.1	[44]
		Smooth stainless steel mesh									~18	
		Poylcarbonate									~14.5	
Rocking cultivation reactor	0.019	Glass-reinfroced plastic	26 ± 2	8	100 [24:0]	11	N/A	8	Modified Basal	*Chlorococcum* sp.	4.26	[30]
		Stainless steel		7		15				*Scenedesmus dimorphus*	0.39	
										*Chlorella protothecoides*	0.11	
										*Chlorella vulgaris*	0.04	
										*Scenedesmus obliqnus*	0.18	
										*S. dimorphus*	0.32	
										*Chlorococcus sp.*	0.53	
Rocking cultivation reactor	0.006	Polystyrene foam	20	N/A	110-120 [24:0]	15	N/A	15	Manure wastewater	*Chlorella* sp.	2.57	[45]
		Cardboard									1.47	
		Polyethylene landscape fabric									0.58	
		Loofah sponge									1.28	
Algal turf scrubber	30	Landfill liner & nylon netting	< 32	7-7.5	outdoor	May and June	93	N/A	Manure wastewater	WW consortium	25	[46]
RAB	0.186	Braid cotton rope	20	8	230 ± 15 [24:0]	3months	N/A	4.8	Petroleum refining wastewater	Mixed culture of algal-bacterial	4.11	[47]
Photorotating biological contactor (PRBC)	1.6	PVC	19-23	N/A	756 [12:12]	60	0.01	2-5	Simulated acid mine drainage	Microbial consortium dominated by *Ulothrix* sp.	0.42	[48]
**Permeated biofilms**												
Filtration photobioreactor	0.0113	5 *µ*m pore size membrane	35	N/A	100	4	0.005	N/A	Synthetic medium	*Chlorella sp.*	13.56	[49]
Single layer vertical plate attached photobioreactor	0.002	0.45 *µ*m cellulose acetate/nitrate membrane	25	N/A	100	8	N/A	N/A	BG 11	*Botryococcus braunii*	6.25	[50]
										*Scenedesmus obliquus*	10.46	
									F/2	*Nanochloropsis*	~5	
										*Cylindrotheca fusiformis*	~5	
Twin layer biofilm photobioreactor (TL-PBR)	0.0005	0.4*µ*m polycarbonate membranes	26 ± 2.5	N/A	1023 [14:10]	31	0.004	N/A	Bold’s basal	*Halochlorella rubescens*	31.2	[51]
Tube-type-twin-layer PBR	0.002	Plain printing paper	26	N/A	67 [15:9]	25	0.003	N/A	Modified F/2	*Isochrysis* sp.	0.60	[120]
										*Nannochloropsis* sp.	0.80	
										*Tetraselmis suecica*	1.50	
										*Phaeodactylum tricornutum*	1.80	
Twin-layer photobioreactor (outdoor)	0.67	Plain printing paper	29	N/A	4-320	25	0.1-0.17	N/A	Modified F/2	*Isochrysis* sp.	4.20	[120]
										*Tetraselmis suecica*	5.1	
										*Phaeodactylum tricornutum*	6.1	
Porous substrate bioreactor	0.0004	Glass fiber filter paper	25	N/A	110 [24:0]	3	N/A	N/A	BG 11	*Anabaena variabilis*	2.88	[52]
Attached cultivation reactor	0.06	0.45 *µ*m cellulose acetate/nitrate membrane	25	N/A	100 [24:0]	7	0.06	N/A	BG 11	*Aucutodesmus obliquus*	9.16	[53]
Phototrophic biofilm reactor	0.125	Polyethylene woven geotextile	21	7	180 [24:0]	40	0.177	N/A	Synthetic wastewater	Wastewater consortium	7	[54]
Horizontal flow lane reactor	0.028	Polyethylene woven geotextile	24	7	200 [24:0]	40	0.2044	N/A	Synthetic wastewater	Wastewater consortium	~7	[54]
Biofilm cultivation reactor	0.09	0.45 m cellulose *µ* acetate/nitrate membrane	17	N/A	200 flashing light	3	0.03	N/A	BG 11	*Scenedesmus dimorphus*	9.13	[55]
Algal biofilm photobioreactor	0.275	Concrete	25 ± 1	8.3	55 [24:0]	35	0.15	N/A	BG 11	*Botryococcus braunii*	0.71	[99]

^a^This parameter indicates the biomass productivity in terms of cell counts.

^b^This parameter indicates the biomass productivity in terms of percentage of colonization area.

N/A: non-available.

All the biomass productivities were reported at the best performing combinations.

### 2.3. Evaluating microalgal biofilm attachment to different substrates

EPS accumulating on the cell surfaces and substrates will uplift the adhesion strength between the cells and substratum. According to Shen et al. (2015), EPS productivity in pure culture medium increased with time and was higher than that in wastewater. They showed that *Botryococcus braunii* produced a maximum EPS concentration of 3770 mg/m^2^ in modified Basal medium (MB) and EPS concentration of 2936 mg/m^2^ in wastewater on the polyethylene foam at day 16 because of sufficient nitrogen and phosphorus supply [13]. However, same algal strain secreted more EPS on glass fiber reinforced plastic in MB at day 16 and recorded a high biomass productivity of 3.2 g/m^2^/day. The authors concluded that the protein secreted from the biofilm cells (51.6%) was the main cause of strong biofilm adhesion on glass fiber reinforced plastic. Similarly, previous researchers cited that protein increased the hydrophobicity of sludge surface, thus improving sludge settling due to the bonding ability of proteins to cations [56]. The relationship between attachment strength and EPS production of *Pseudomonas* sp. and *Amphora coffaeformis* was also investigated by Becker, and findings displayed that some proteins adhered better to hydrophobic than to hydrophilic materials as nonpolar attachment structure form localized hydrophobic binding sites. Strong adhesion usually consists of huge compounds in which their macromolecules can come in close contact with a wide range of surfaces [11].

There were scientific reports on the isolation and partial chemical analysis of EPS from the microalgae to date, especially extracellular polysaccharides because it is a major component in the biofilm matrix [57–59]. In a biofilm, the functional groups of exopolysaccharides depict strong affinity toward different dissolved organic matters and metal ions [60]. Polysaccharides can interact with ions and proteins via various interaction forces, such as electrostatic attractive forces, repulsive forces, hydrogen bonds, van der Waals interactions and ionic attractive forces [61]. EPS polysaccharides in microalgae can be constituted of more than ten monosaccharides as presented in Table 3. Among those monosaccharides, glucose concentration was the highest as compared with the others [62]. In the biofilm cells of *Amphora* sp., glucose was the most abundant followed by galactose, mannose and rhamnose with mole percentage over 10% and xylose, fucose and N-acetyl-D-glucosamine in mole percentage less than 5% [57]. In the same study, the biofilm cells of *Stauroneis* sp. were also found to be abundant in glucose with mole percentage of 60.98% followed by mannose and galactose, but other monosaccharides were barely detected. The ability of *Amphora* sp. to form biofilms was eventually claimed to be stronger due to the diversity of monosaccharide composition in the EPS.

**Table 3.** Classification of monosaccharides in microalgal EPS. (Adapted from [63])

Monosaccharides	Example
Neutral sugars	arabinose (Ara), ribose (Rib), xylose (Xyl), glucose (Glc), galactose (Gal), mannose (Man), quinovose (Qui), fucose (Fuc), rhamnose (Rha)
Uronic acids	glucuronic acid (GlcA), galacturonic acid (GalA), mannuronic acid (ManA)
Amino sugars	Glucosamine (GlcN), galactosamine (GalN), mannosamine (ManN)
Uncommon sugars	3-deoxy-D-manno-2-octulosonic acid (Kdo), Neuraminic acid (Neu)

In marine fouling diatoms, multiple studies demonstrated that acidic sugar monomers such as uronic acids and sulfonic acids which are important constituents of TEP in the mucous matrix contributes towards acidic binding sites that act intercellular glue for cells aggregation [31]. Sulfated exopolysaccharides in diatoms are able to give awide range of monomer linkage combinations and provide huge polysaccharide diversity, thus offering protection from desiccation and cation exchange [64]. For instance, approximately 20.85% of the EPS from biofilm cells of *Navicula subinflata* were made up of uronic acids, pyruvate, hexoxamines, methylpentoses and sulfate that lead to stronger biofilm formation ability [62]. These components are often found attached to the polysaccharide chains as side groups and contain carboxyl, amino and sulfate groups [60]. Uronic acids enriched in diatoms confer negative surface charges and acidic properties to the EPS, exhibiting high copper binding capacity [65]. As cited by Bhosle etal. (1995), this unique property of uronic acids in the microalgal EPS offers ecological benefit to grow the diatoms on surfaces coated with toxic antifouling compounds such as cuprous ions. Another adhesive polymer, the proteins, exist in considerable amounts in environmental biofilms and normally classified into enzymatic proteins and structural proteins [66]. Enzymatic protein is crucial in sustaining the cell metabolism by acting as an external digestive system to degrade the excess EPS components, while structural proteins determine the attachment process of the microorganism to the substrates [17]. Protein breaks down huge-molecular-weight EPS products into smaller molecules to be consumed by the cells. Some proteins are electron donors or acceptors in the redox reactions on biofilms and render anionic properties to the EPS, contributing acohesive energy to the EPS matrix. In this case, the negative charges of the proteins are associated with the presence of amino acids such as aspartic acids [67]. Results of total protein–carbohydrate ratios obtained from the lab-scale experiments with predominance of protein in the EPS depicts greater biofilm stability and robustness such as in *Amphora* sp [11]. EPS molecules can overcome the electrostatic repulsion forces between the cells and substrates via polymer bridging, while attractive electrostatic forces between charged proteins in EPS provides cohesive stability to the biofilm [68]. However, as compared with polysaccharide analysis, information about the effect of protein components to the adhesion strength is scarce, especially the contribution of the amino acid profiles in microalgae EPS toward biofilm formation.

This review on EPS composition focuses only on polysaccharides and proteins because there is not much information available for other components, such as lipids and nucleic acids. Although non-sugar components are typically having a lower concentration than polysaccharides, they do play an active role in the construction of the biofilm matrix. Hence, more detailed research should be encouraged to probe into the contribution of those components, especially in their molecular mechanisms. Both polysaccharides and proteins in microalgal EPS have varying molecular weights depending on the degree of polymerization, with molecular weights ranging from 2 Da to 7,000 kDa due to the presence of glucuronic acid and sulfate groups [69]. It is noted that molecular weight was highly associated with the surface charge density as high-molecular-weight organic matters act as a flocculant aid, while low-molecular-weight organic matters increase the negative charge at the particle surfaces [70]. Hence, it is encouraged to predict the biofilm adhesion onto different substrates by analyzing three major aspects of microalgal EPS: (1) biochemical elemental composition, (2) molecular weight distribution, and (3) hydrophobicity.

## 3. Factors affecting production of extracellular polymeric substances

Biofilm adhesion depends completely on the EPS productivity by microalgae as the interactions involved between the macromolecules alter the structural integrity of the biofilm structure. Though archival literature revealed a lot about the impacts of attachment materials and microalgal strains selection on the biofilm adherence, some pivotal operational characteristics such as nutrient availability, light intensity, CO_2_ concentrations, pH and temperature have been identified to play their roles as well. An in-depth comprehension of various strategies to optimize EPS production might reduce the bottlenecks in large-scale microalgal biofilm-attached cultivation systems, as highlighted in this section.

### 3.1. Nutrient starvation

Essential inorganic nutrients such as nitrogen, phosphorus, sulfur, carbon and iron have notable impact on metabolism of microalgae cells, as nutrient limitation is a promising strategy to regulate the biochemical pathways of the microalgal cells [71]. Bounded EPS from nitrogen and phosphorus starved *Chlorella* sp. ADE4 had higher carbohydrate and protein amounts as compared with the control condition, in which the nitrogen and phosphorus was sufficient [72]. Unfavorable growing conditions, such as a suboptimal nitrogen-to-phosphorus ratio in the wastewater, induced EPS production from algae to aid in nutrient absorption from the environment, resulting in higher protein fractions in the EPS [73]. Similarly, Boonchai et al. (2014) found out that in starved culture, the protein content in soluble EPS (4.54%) was more than that in bounded EPS (2.33%) because microalgae in nitrogen-deficient condition stored nitrogen outside their cells in the EPS form instead of inside the cells.

However, there were several studies showing that nutrient depletion did not favor firm biofilm bonding, as the biofilm growth kinetic and sloughing were significantly affected by the levels of nitrogen and silicon [74]. When nitrogen (<1 mg/L) and silicon (<0.01 mg/L) were flushed from media, the biofilms sloughed from the substrates and ceased to grow, while a non-starved culture of *S. obliquus* biofilm grew well without sloughing for 26 days. Biofilm grow stopped under nitrogen starvation because the cells were unable to produce essential metabolism components such as amino acids, nucleic acids and proteins for cell replication, while silicon depletion caused the diatoms to stop producing the frustule exoskeleton [75,76]. For microalgal biofilm with the presence of bacteria, nutrient starvation induces the bacteria to release alginate lyase that degrades the EPS matrix bonding of the algae cells to the substratum, eventually causing biofilm sloughing. Hydrolysis of EPS may also occur during nutrient starvation for the utilization of the biomolecules in the biofilm matrix [74].

It is a well-established fact that algal cultures expose to nutrient starvation stresses can substantially trigger lipid accumulation response. An autotoxin will be released by starved algae to inhibit cell division, but photosynthesis process will be kept constant for carbon assimilation to enhance lipid yield. These neutral lipids are formed as dense lipid bodies in the cytoplasm as a result of the conversion of assimilated carbons in polysaccharides, proteins and other internal biomolecules [77]. However, *Nitzschia palea* and *Scenedesmus obliquus* biofilm did not show higher lipid accumulation as compared to planktonic cultures under nutrient deficient conditions for 6–7 days, since the EPS matrix of algal biofilm was turned to be a nutrient reservoir, making the biofilms notoriously resilient to environmental stresses [74]. Higher cell density and EPS amount in mature biofilm tend to signify higher nutrient utilization rate as the EPS also aid the attachment of other non-microalgae organisms such as mineralizing bacteria which degrade the EPS, lowering the lipid concentration of the cells within the biofilm [78]. Theoretically, lower EPS and bacterial concentrations in the biofilm result in greater lipid accumulation within the cells.

### 3.2. Light attenuation

Light is one of the most important energy sources for microalgal biofilm development on different surfaces and affects the EPS yield. The EPS produced by *Nostoc* sp. at 80 μE/m^2^/s (206.20 mg/g DW) was higher than that at 40 μE/m^2^/s (155.49 mg/g DW) [79]. As reported by the authors, higher light intensity increased carbon dioxide fixation rate, nitrate assimilation rate and metabolism rate in the cells. Nevertheless, Ge et al. (2014) found out that higher light intensity barely affected the monosaccharide composition but resulted in 1.3-fold increase in protein content, suggesting that light attenuation regulates not only biofilm growth or adhesion but also EPS accumulation. Identification of the optimum light intensity range is a must for microalgae biofilm growth as both low and extreme light exposure will cause undesirable responses toward the cells due to photo-inhibition and photo-oxidation [71]. Exposure of algae to photon flux density beyond the saturation point causes disruption of chloroplast lamellae and inactivation of enzymes in carbon dioxide fixation [80].

Even though extensive number of studies have been conducted on the effect of light intensity on EPS productivity, the results obtained varies between microalgal species and culture conditions. As an example, *Botryococcus braunni* CCALA 778 in outdoor cultivation showed the highest EPS productivity at 0.29 g/L/day under 2000 μE/m^2^/s (16:8 light-dark cycle) in resemblance to the summertime, while *Porphyridium cruentum* cultured in flat plate photobioreactors obtained 0.095 g/L/day of EPS polysaccharide production at 80 μE/m^2^/s (18:6 light-dark cycle) [81,82]. Moreover, biofilm sloughing issue in illuminated attached cultivation systems must also be taken into consideration, especially on thick biofilm which has high chances of light limited conditions in the lower biofilm layer [83]. Bacteria and other non-photoautotrophic materials will dominate the layer, restricting the mass transfer of nutrient to the bottom cells and eventually end up with biofilm exfoliation, when the biofilm thickness has reached its peak [84]. In order to aim for higher biomass productivity, it is crucial to maintain the biofilm at an appropriate thickness by re-harvesting, allowing the bottom cells to serve as an inoculum for the next growth cycle [85].

Light spectrum has also been recognized to be critically influence the microalgal EPS composition as results proved that both blue (400–500 nm) and red (600–700 nm) lights effectively increased the polysaccharide production of *Porphyridium cruentum* [80]. Findings were consistent with *Chlorella* sp. biofilm (filtration membranes as substrates) cultured under blue (440–500 nm), green (500–550 nm) and red (610–650 nm) lights, in which higher polysaccharide accumulation was promoted than that of white (400–750 nm) light [86]. In the same study, both *Nannochloropsis oculata* and *Chlorella* sp. accumulated the greatest lipid amount under blue light as this specific light wavelength stimulated the enzymatic activities of carbonic anhydrase and ribulose bi-phosphate carboxylase/ oxygenase to form triglycerides, but protein content extracted from microalgae under four different lights remained similar. It was also discovered that white, blue and purple lights were able to give higher biofilm formation of *Chlorella vulgaris* than red, yellow and green lights alone [87].

### 3.3. Carbon dioxide concentration

For algal biofilm growth, high CO_2_ concentration will increase the mass transport rate and provide better growth kinetics. For instance, when the CO_2_ was uplifted in the gas feed from 2% to 13%, total EPS excretion of *Chlorella vulgaris* was higher, and the results obtained were in agreement with its maximum CO_2_ bio-fixation rate at 0.98 CO_2_/L/day for 13% CO_2_ [88]. However, when the culture of microalgae cells is aerated with high CO_2_ concentration, part of the carbon is consumed via photosynthesis, while the remaining carbon is converted to carbonic acid that acidifies the culture medium, affecting the cell metabolism [71]. To justify this, the pH values decreased from 8.7 to 6.0, when the CO_2_ level was increased from 0.03% to 10% in the feed gas flow [89]. Drastic pH changes in the medium will undoubtedly lead to enzymes deactivation involved in the photosynthesis process, halting the cell growth and EPS production. Hence, optimum pairing of pH, light attenuation and CO_2_ concentration must be considered for higher biofilm stability onto the substrates.

Moreover, interaction effects between CO_2_ and light intensities have to be taken into account as it is possible that the microalgal biofilms receive insufficient photons to assimilate the excess inorganic carbon obtained from the gas feed, causing carbon saturation at the system [19]. As CO_2_ concentration increased, *Ettlia* sp. biofilm thickened with top biofilm layer experiencing high light stress, resulting in triacylglycerol accumulation as carbon storage. Meanwhile, the bottom biofilm layer only encountered biomass increase with negligible lipid production but ended up with starch accumulation as a carbon reservoir instead [90]. This phenomenon was presumably attributed to the muted effect described by Schnurr et al. (2016), whereby a shading effect was rendered by the upper layer of the biofilm, thereby restricting the triacylglycerol production in the cells at the bottom layer [91].

### 3.4. pH

pH is another key factor in establishing the microalgal biofilms adhesion and may vary in different biofilm depth [8]. Between the pH range of 1–11, the surface potential of microalgal cells is always negative [92]. This is due to the components of cytoplasmic membrane, which impart the surface electrical property of microalgae. Zhang et al. (2011) explained that small amounts of glucide and micro-nucleic acid are present in the membrane bilayer lipid. These amino acids possess negative charge under normal condition, thus rendering the cells charge negativity. Meanwhile, the cell surface hydrophobicity can be greatly affected by the presence of amino acids, such as alanine and tyrosine, which contribute to stronger hydrophobicity.

However, surface electric potential of cells decreased with pH when the pH was less than 7 because the concentration of hydrogen ion increased. The carboxyl dissociation on protein was inhibited, while the amino dissociation was enhanced, weakening the electronegativity of protein molecules and directly increased the adhesion to the substrates [83]. If pH is higher than 9, microalgae cells tend to release more EPS to shield themselves from extreme pH changes, thus resulting in higher attachment of diatoms, as seen in *Nitzschia amphibia,* to both titanium and glass under alkaline range (pH > 7) [8]. The optimal pH for biofilm growth varies among microalgal species. As in the case of *Chlorococcum* sp. on glass fiber-reinforced plastic, pH 8 was the optimum value for its biofilm growth [30]. In short, microalgal biofilm attachment is favored at near neutral pH between 6 and 9. The cells hydrophobicity will increase with pH and the dissolved CO_2_ concentration was at equilibrium with the pH without causing extreme acidic environment [19].

### 3.5. Temperature

Variation in temperature influences the metabolism rate of the microalgae cells. Each microalgae species has different temperature requirements, but generally, the optimum temperature range for algae cultivation is 20–25°C [83]. For example, *Cylindrotheca closterium* had accumulated a total EPS of about 15 μg/mL at 25°C [93]. Besides, sticky exopolysaccharides were proven to increase with temperature till it reached a peak and decreased at higher temperature for different marine microalgae such as *Thalassiosira pseudonana, Pseudonitzschia fraudulenta, Skeletonema marinoi, Isochrysis galbana* and *I. aff. galbana* [94]. These muco-polysaccharides are usually produced by marine microalgae in large amount and promote aggregation mechanisms, resulting in strong biofilm adhesion [31]. Low temperature (<16°C) will slow down the algae growth, while high temperature (>35°C) will be lethal to some species [95].

An abrupt temperature change generally creates disproportion between the photosynthetic energy supply and energy consumption within the Calvin cycle inside the algal cells. Inherent physiological functions of the photosynthetic apparatus will be altered due to temperature variation, which is sometimes referred to as photosynthetic temperature acclimation [96]. At lower temperature, carboxylase activity is reduced, but there will be an oversupply of energy if light remains unchanged. Under elevated temperature, both *Microcystis aeruginosa* and *Scenedesmu acutus* acheived higher photosynthetic rates, but lower respiration rates and smaller cell size [97]. The warming phenomenon increases the demand for resources and causes the cells to shrink in order to compensate for the imbalance between catabolic and anabolic processes, thus lowering metabolic activities [98]. As compared with suspension culture systems, microalgal biofilm cultivation systems are more susceptible to temperature fluctuations because there is lesser amount of water in the system to act as temperature buffer [99].

## 4. Mathematical modeling for algal adhesion prediction

**Figure 3.**
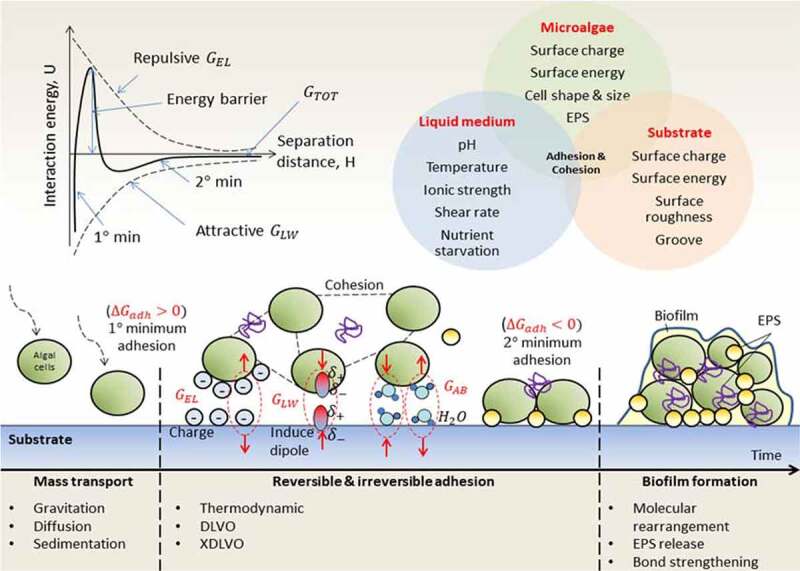
Application of the interaction forces during biofilm formation. (Modified from [106,107])

Microalgae have strong tendency to adhere to different surfaces with different degree of hydrophobicity [100]. When both the microalgae and substrates are in an aqueous environment, different transport mechanisms such as Brownian motion, gravitation, diffusion, convection or intrinsic motility of microalgae will be involved for cell transportation to the substrate surfaces. The physico-chemical interactions can be divided into adhesion to substrates, co-aggregation between microalgal pairs and co-adhesion between sessile and planktonic microorganisms of different strains [101]. In fact, all the interactions forces are originating from the same fundamental forces likes Lifshitz-van der Waals forces, electrostatic forces and acid-base interactions [102]. According to Bos et al. (1999), once the macroscopic interaction forces allow two surfaces to interact, complementary stereo-chemical groups will attract each other even at a distance in few nanometers only. All the adhesive interaction models are evaluated based on the contact angles measured by different probe liquids and surface potential of the cell surfaces and substrate surfaces. Roughness and porosity of the materials have no bearing on the modeling. Hitherto, there were lots of remarkable studies being published about the surface tension of different microalgal strains and substrates to examine the free adhesion energies [103–105]. In this section, some significant physic-chemical approaches toward the adhesive interactions are summarized and presented in Figure 3.

### 4.1. Thermodynamic approach

In this approach, the interacting surfaces are assumed to be physically in contact with each other under thermodynamic equilibrium. This approach determines the changes in the total interfacial free energy ($\Delta G_{adh}$) and does not include electrostatic interactions. It is calculated using the Lifshitz-van der Waals-acid base (LW-AB) approach according to the extended Young’s equation [108]. The attachment of the cell is thermodynamically favorable if $\Delta G_{adh}$ is negative, while adhesion is unfavorable when $\Delta G_{adh}$ is positive [101]. For example, greater adhesion strength was indicated for *Botryococcus sudeticus* on both hydrophilic glass (−9.2 mJ/m^2^) and hydrophobic indium-tin-oxide-coated glass (−65.9 mJ/m^2^), especially when both the surfaces of cells and substrates are hydrophobic, which can result in huge acid-base attraction [104]. This result was only valid in close contact as it did not include long range electrostatic interactions [101]. Moreover, another study cited that this model analysis fitted well to the diatom cell attachment of *Navicula jeffreyi* onto polytetrafluoroethylene, polyethylene, stainless steel and polyamide-nylon [103]. Besides, adhesion of *Chlorella vulgaris* grown in microelement-limited medium was found to favor propyltriethyoxysilane-modified glass slides with a total free adhesion energy of −4.8 mJ/m^2^ but its prediction did not able to provide a complete description of the algal adhesion to solid surfaces [105]. Hence, it was confirmed that not all the experimental algal adhesion studies can be well predicted with this theoretical approach, but it definitely give merits to the first and most fundamental adhesion prediction [109].

### 4.2. Derjaguin, Landau, Verwey, Overbeek (DLVO) approach

In this approach, microalgal adhesion is described as a balance between attractive Lifshitz-van der Waals (LW) and repulsive or attractive electrostatic interactions (EL). This model prediction needs the input of zeta potential of all the interacting surfaces as it takes into account the electrostatic forces. This model studies the interaction energies based on the distance decay, as well as determines the extent of reversible adhesion. Ionic strength affects the magnitude of electrostatic interactions due to the shielding of surface charges but do no affect the Liftshitz-van der Waals attraction [101]. This model suggests that negative total interaction energy ($G^{TOT}$) indicates adhesion, while a positive indicates repulsive cell-substrate interaction. For instance, DLVO fitting model showed that *Chlorella vulgaris* will adhere to the glass at a separation distance of 13.6 nm with a local energy minimum of −39.4 kT and that was due to the weak secondary adhesion. This type of adhesion possessed very small attractive interaction when the cells were still far away from the surface. On the other hand, stronger primary adhesion was found for the same species with indium-tin-oxide coating glass at −3,868 kT globally [104].

### 4.3. Extended DLVO (XDLVO) approach

This approach is the extended version of DLVO, which considers the contribution of the acid-base interactions (AB) in addition to LW, EL and Brownian motion forces. It is considered as a combination of the thermodynamic and classical DLVO model. Acid-base interactions are based on electron transfer between polar components in the aqueous solutions. It can be hydrophobic attraction or hydrophilic repulsion. As suggested by this model, the main driving force for hydrophobic attraction is the cohesion energy of water molecules due to the hydrogen bonding. However, acid-base force is short-ranged, so close distance between the interacting surfaces (<5 nm) is necessary for the force quantification to be valid [101]. This model indeed predicted the existence of interaction energy valleys for algal adhesion. To illustrate this, the deepest energy valley had verified that *Chlorella vulgaris* cultured in complete mineral medium attached the best with 3-aminopropyltriethoxysilane modified glass slides [105]. Introduction of acid-base interaction in this model might change the adhesion mode as predicted by classical DLVO model [104]. As mentioned by Ozkan and Berberoglu (2013), *Botryococcus sudeticus* was expected to contact with indium-tin-oxide-coated glass at a larger attractive force by changing its secondary adhesion to primary minimum adhesion. This model was also used to predict the bubble-particle adhesion in air flotation process for *Anabaena variabilis* and *Chlorella vulgaris* [110].

### 4.4. Comparison between colloidal approaches

Cell adhesion toward surfaces can be described by employing three main colloidal approaches as stated beforehand. Nonetheless, there were indications from previous literature of possible conflicts between theoretical assumptions and genuine experimental findings, underscoring the need for vigilance. Being the simplest adhesion model, thermodynamic approach is limited as it is only able to elucidate the cell attachment process in protein-free environment. Moreover, this approach solely depends on the surface free energies of the entities and neglects the influence of electrical charge and with the assumption that there is no chemical bonding between cells and substrates [111]. Although DLVO theory considers the effects of attractive van der Waals forces and repulsive double layer forces, it is illusory in the sense that projection can only be accounted by using fitting parameters to accommodate surface potentials or charges. Unsatisfactory DLVO results frequently indicate substantial deviation even in small-scale studies due to lack of measurement of hydration forces, hydrophobic or water structural forces. Furthermore, DLVO theory only appears to work well in low salt concentrations (electrostatic forces dominate) by ignoring the dispersion forces acting on the ions at different biological salt concentrations, thereby eventually leading to flawed estimations [112].

XDLVO model was extensively used for adhesion prediction as it provides better prediction than DLVO [113]. However, the cell adhesion is also affected by the fluid flow rate and the shear forces in the cultivation system as well, but these are not considered in XDLVO model. Considering these facts, the three approaches are only able to provide a rough estimation on the biofilm adhesion based on the dimension, hydrophobicity and zeta potential of cells and substrates. Besides that, changes of EPS composition influenced by different surface characteristics affect hydrophobic protein proportion, and this may contribute to different type of adhering mechanisms. As an example, hydrophilic materials with good liquid-holding capacity are preferable for *Chlorella, Chroococcus, Chlorosarcinopsis, Synechococcus* and *Scenedemus,* which are easily immobilized by the hydrophilic polymers [114]. Moreover, biofilm adhesion can also be affected by surface roughness, but several reports have shown otherwise due to diversified nature of microorganism studied [113,115]. High adhesion forces have been shown to improve cell adhesion but are sometimes detrimental to the cell’s survival after attachment to the substrates because these forces might induce stress to the cells, limiting their ability to further replicate [116]. Cell adhesion is completely a complicated species- or even strain-dependent process involving different physical structures and biomolecular components of the cell surfaces. Therefore, these prediction models can only provide a rough estimate of cell attachment tendency, and a successful verification with experimental adhesion studies is still required. However, they are still valuable to serve as a fundamental guide in algae strain or supporting material selection studies.

## 5. Future prospects and challenges

The EPS extracted from microalgal biofilms are complex mixture of biopolymers that functions as scaffolds for micro-consortia and mostly contributed to biofilm stability. Since microalgal biofilm cultivation requires biofilm formation for growth, it is vital to look into the makeup of EPS and their role in immobilized microalgae cultivation. Although, at present, EPS extraction studies has been extensively carried out, the range of techniques required to extract known biopolymers highlight a broad choices among biofilm researchers. In other words, there is no consensus on a standardized EPS extraction method, leading to different extraction efficiencies and discrepancies in the biopolymer fractions [66]. In view of this, EPS results acquired from different investigations were not comparable due to the lack of a universal baseline. Similar situation was also observed for chemical/mechanical based extraction methods, such as mechanical pre-treatments, acidification, enzymatic hydrolyze, alkalinization and heat treatments being commonly invoked in polysaccharides extraction or cell lysis in cytosolic protein extraction [117]. As of now, any extraction employed has the potential to cause structural damage to the cell integrity and this will lead to contamination of the EPS. Hence, profound understandings of the risk involved during the EPS assessment are needed to guide new procedural breakthrough for a more accurate intra- and extracellular cellular evaluation. A standardized selection guide on the extraction methods is needed and can be adjusted accordingly to the microalgae species and strains.

Despite the intricate nature of EPS in multi-species biofilm, researchers tend to conserve the role of EPS across all types of biofilms. However, it is a fact that the role of EPS are highly attributed to their unique biophysical and structural features such as the presence of different types of functional groups, chemical bonding types, and characteristics. The myriad types of EPS fraction and its presence deserved to be look into and studied. These EPS can be quantified with analytical techniques owning greater reliability such as nondestructive fluorescence lectin-binding analysis that uses lectins (or glycoproteins) to specifically bind to the polysaccharides without altering the structures [118]. Due to the limitation offered by the available type of fluorescent labeled probes, promising combinations of confocal laser scanning microscope and magnetic resonance microscopy enabled identification of biofilm structure, metabolic pathway and metabolite concentration of particular molecular groups within the biofilm biomass [119]. The functional activities of the polysaccharides are closely associated with their distinct structural orders, including: (a) molecular chain flexibility due to glycoside bonds, (b) carbon skeleton nature (either homo-polysaccharide or hetero-polysaccharide), (c) molecular weight and mass distribution, and (d) functional groups and position [78]. DNA-sequencing methodologies in conjunction with bioinformatics analyses are recommended for detailed EPS characterization across environmental biofilms to reflect their individual representations of genome annotation and pathway modeling [117]. Molecular mechanism of the biofilm matrix development can be then established to predict and manipulate the EPS production in algal-biofilm cultivation systems.

It is often encouraged to develop longer duration pilot or full-scale biofilm-based cultivation systems for higher biomass and EPS yield in the wastewater sectors [7,99,120]. However, EPS are naturally entangled at molecular level and own high reversibility in their structural changes, complicating the isolated studies of those specified biopolymers. Therefore, full-scale systems might not be an ideal starting point for comprehensive EPS characterization [83]. The physiological behavior of microalgae and dynamics of EPS formation in reaction to biological changes in the biofilm remain debatable. As summarized in this work, the biofilm adhesion strength between different strains and materials varies in a vast range of model systems. Better resolution of the microalgal biofilm EPS characterization should be made available prior to any huge-scale setups. Selection guidelines can be set up for different microalgal strains and substrates by incorporating the physiological properties such as pH, temperature, light regime and flow rate to act as a reference point. It will be an optimum strategy for biofilm control as the biofilm growth greatly relies on the EPS production.

## 6. Conclusions

Microalgal biofilm-based cultivation systems have undoubtedly gained both the scientific and industrial partnership globally, which accelerate the biotechnological development of the cultivation system. Its immense potential leads to vast range of fundamental-based microalgal biofilm researches, such as role of EPS production, impact of operating parameters during cultivation and substrate surface properties. There is no biofilm without EPS matrix. A firm irreversible adhesion occurs when the EPS secreted by the sessile microalgae stimulate the adhesion of other suspended microalgae, together anchoring onto the substrates. EPS production has been verified to strengthen the biofilm adhesion and it can be benefited from changes in nutrient availability, light intensity, CO_2_ concentration, pH and temperature. When the algal cells are brought into close contact with the substrate surfaces, fundamental interaction forces will take place to speed up the biofilm attachment. Therefore, these understanding would serve as a crucial role in accelerating the innovation of novel biotechnologiesand provided a holistic approach toward microalgae cultivation.

## Research highlights

- Microalgal-biofilm cultivation systems enhance biomass productivity by at least 20%.
- Extracellular polymeric substances (EPS) are important in biofilm adhesion.
- Culture conditions significantly affect EPS production.
- Cell-substrate attraction forces stimulate cell aggregation.
